# Effects of Continuous Epidural Injection of Dexamethasone on Blood Glucose, Blood Lipids, Plasma Cortisol and ACTH in Patients With Neuropathic Pain

**DOI:** 10.3389/fneur.2020.564643

**Published:** 2021-01-13

**Authors:** Xiao-hui Liu, Yu-min Du, Hai-jing Cong, Guang-zhao Liu, Yu-E Ren

**Affiliations:** Department of Pain Management, The Second Hospital of Hebei Medical University, Shijiazhuang, China

**Keywords:** dexamethasone, epidural space, blood glucose, cortisol, ACTH

## Abstract

**Objective:** To study the effects of continuous epidural injection of dexamethasone on blood glucose, blood lipids, plasma cortisol, and adrenocorticotropic hormone (ACTH) in patients with neuropathic pain.

**Methods:** Thirty patients with cervical spondylotic radiculopathy, lumbar disc herniation, herpes pain or postherpetic neuralgia were randomly divided into three groups and were treated with different doses of epidural injection of dexamethasone (Group S with a concentration of 25 μg/mL; Group M with a concentration of 50 μg/mL; Group L with a concentration of 100 μg/mL). Epidural catheterization placement was guided by computed tomography (CT), and was connected to the analgesic pump for 10 days. Visual Analog Score (VAS), fasting blood glucose (FBG), total cholesterol (CHOL), triglyceride (TG), 2 h postprandial blood glucose (2hPG) and the concentrations of cortisol, ACTH were measured before injection (T_0_), 2, 4, 6, 8, and 10 days during injection (D_2_, D_4_, D_6_, D_8_, D_10_), and 7, 14, 21, 28 days (W_1_, W_2_, W_3_, W_4_) after injection.

**Results:** During and after the treatment, VAS score was significantly decreased, and group M and L had the lowest VAS score. The concentrations of cortisol and ACTH were significantly lower during the treatment, but all of them recovered to the normal level after stopping the injection. The treatment did not affect the CHOL and TG concentrations.

**Discussion:** Epidural injection of dexamethasone at the concentration of 50 μg/mL is recommended for patients with neuropathic pain because of its good analgesic effect and less adverse effect on blood glucose, plasma cortisol, and ACTH.

## Introduction

Glucocorticoids can inhibit the production, release, and activation of many inflammatory mediators and cytokines, stabilize the lysosomal membrane, and reduce the damage of these inflammatory mediators, cytokines. Epidural steroid injection (ESI) has a good therapeutic effect on neuralgia related diseases, such as cervical spondylotic radiculopathy, lumbar disc herniation, and herpes zoster and postherpetic neuralgia (1, 2), it can rapidly reduce local inflammation, relieve or eliminate neuropathic pain by selectively acting on the lesion and keeping a relatively high concentration, clinical practice is considered effective (3). ESI has been endorsed by the North American Spine Society as an integral part of non-surgical management of radicular pain from lumbar spine disorders (4).

Glucocorticoid drugs have a wide range of effects, and have a great impact on the metabolism of carbohydrate, fat, and protein; they also have an impact on the hypothalamic-pituitary-adrenal axis (HPAA), which can inhibit the synthesis and secretion of cortisol and adrenocorticotropic hormone (ACTH) through feedback action on the hypothalamus and pituitary. ESI is different from systemic medication with a very small dose injected directly into the lesion, which may reduce the systemic side effects of the hormone. Initially, clinical scientists only paid attention to the local side effects of ESI, such as the risk of infection and hyperplasia of fat tissue in the epidural space (5). With the increasing clinical application, more attention is focusing on the systemic effects of ESI. Several studies have shown that single or multiple epidural injections of steroids can temporarily increase fasting blood glucose level or postprandial glucose (6, 7), and can cause a transient adrenal dysfunction (8).

Cervical spondylotic radiculopathy, lumbar disc herniation, herpes zoster and postherpetic neuralgia are common neuropathic pain which can greatly decrease the patients' quality of life. In the United States, the total cost related to low back pain (LBP) exceeds $100 billion per year (9). About two-thirds of people will experience neck pain, especially in middle age (10). Epidural steroid injections are widely accepted to work better for neuropathic pain (11).

In clinical practice, continuous epidural injection of steroids is often required to achieve satisfactory analgesic effects, such as in patients with herpes zoster and postherpetic neuralgia (12). However, it is still largely unknown whether continuous epidural injection of corticosteroids can cause systemic adverse reactions. The aims of this study were to explore whether continuous epidural injection of corticosteroids can affect blood glucose, blood lipids, plasma cortisol and the concentration of adrenocorticotropic hormone (ACTH) among patients with neuropathic pain, and to investigate which dose of dexamethasone might have a good analgesic effect but with less adverse effect.

## Materials and Methods

### Study Participants

This study was approved by the ethical committee of the Second Hospital of Hebei Medical University (2017-R017). Patients who met the following inclusion criteria were included: patients who experienced cervical spondylotic radiculopathy, lumbar disc herniation or herpes zoster and postherpetic neuralgia (except cranial nerve), Visual Analog Score (VAS) ≥ 5, age between 30 and 80 years, the body mass index ranging from 18.5 to 30 kg/m^2^, signed informed consent. Exclusion criteria included a history of corticosteroid treatment during the past 2 months, contraindications for epidural puncture, chronic diseases (i.e., diabetes mellitus, hyperlipidemia, hypothyroidism, renal disease, liver disease), a history of any type of adverse reactions to steroids or local anesthetics, allergy to contrast agents and pregnancy, lactating, or intending to become pregnant. Thirty patients were prospectively recruited in the study between 1st March 2017 and 31st October 2017. Patients were randomly assigned to three groups (*n* = 10): S group, M group and L group. In addition, a sham group is not needed in our study, because ESI is very effective and our main aim is to ensure the patient's treatment effect.

### Epidural Analgesia

**Figure 1:**
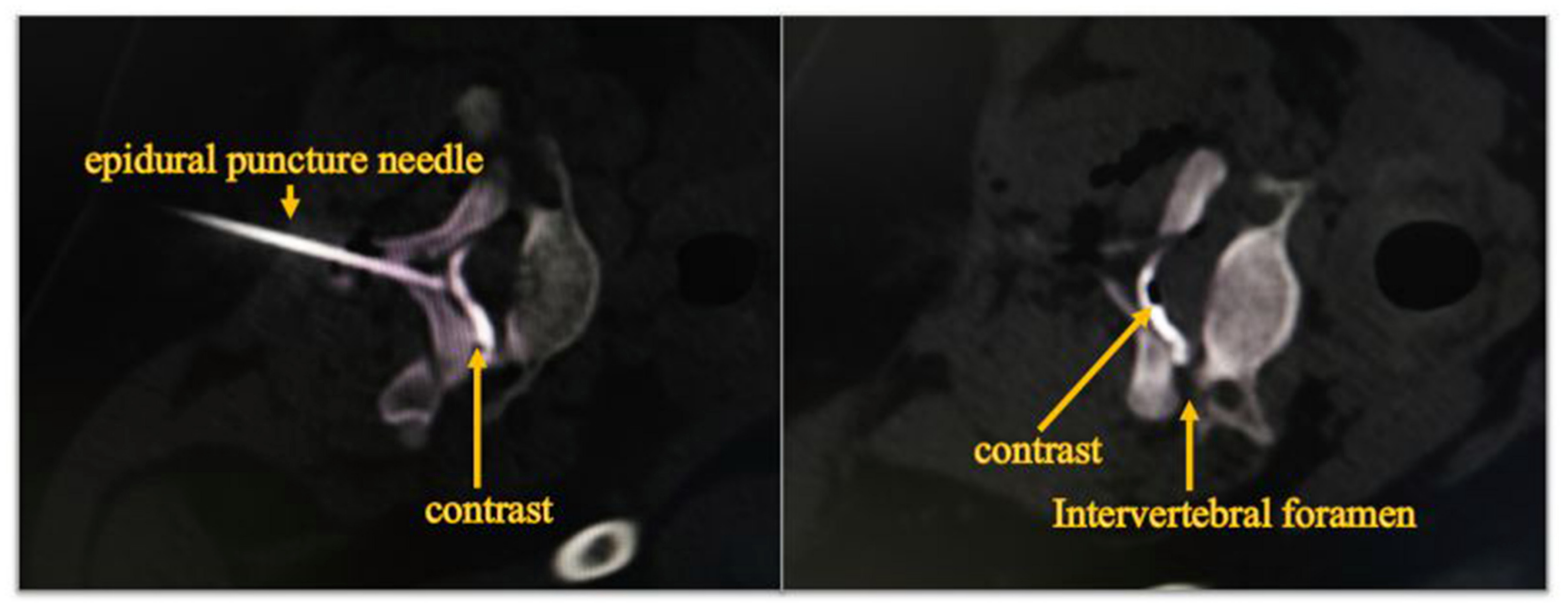
Diffusion of contrast agent in epidural space at CT fluoroscopy. The image shows that the epidural puncture needle reaches the epidural space, and contrast injected into the epidural space spreads outward along the nerve root canal.

We first determine the location of the nerve lesion, and then put the catheter into the affected nerve site by epidural catheterization. Epidural injections were performed under CT guidance in order to confirm the exact localization (Figure 1). The analgesic pump was connected later to keep the continuous injection of dexamethasone, bupivacaine, fentanyl and saline mixtures. The mixtures consist of bupivacaine 0.075% and fentanyl 2 μg/mL with an injection speed of 2 mL/h. The only difference among the three groups was the doses of epidural dexamethasone. Group S was treated with dexamethasone concentration of 25 μg/mL, group M with a concentration of 50 μg/mL and group L with a concentration of 100 μg/mL. A new analgesic pump was replaced every other day. Tenderness, redness, leakage as well as the depth of catheterization at the puncture site had were recorded. The analgesic pump was removed after 10 days of injection.

### Observation Index

#### Analgesic Effect

The VAS of patients before injection (T0), 2, 4, 6, 8, 10 days during the injection (D2, D4, D6, D8, D10) and 7, 14, 21, 28 days (W1, W2, W3, W4) after injection was recorded. By using a 10-cm ruler, “0” represents none pain on left-hand end, and “10” represents an extreme amount of pain on right-hand end. The VAS score was determined by measuring in millimeters from the end of the line to the point that the patients marked.

#### Laboratory Index

Fasting blood glucose (FBG), total cholesterol (CHOL), triglyceride (TG), plasma cortisol (COR), ACTH concentration, 2 h postprandial blood glucose (2hPG) at each time point above. FBG and 2hPG were measured by the oxidase method, CHOL by enzymatic colorimetry and TG by colorimetric method. COR and ACTH concentration were tested by chemiluminescence analysis.

#### Adverse Events

Occurrence of adverse reactions such as itching, nausea and vomiting, urinary retention, catheter loss, catheter fracture, and catheter-related infection.

### Statistical Analysis

Statistical analyses were performed using the statistical software SPSS19.0. The sample size was calculated using the G^*^power software. We assumed that the effect size for the main outcome of VAS was 0.45, and we needed a total of 30 patients if we would like to achieve 80% power for the detection of difference. The data were presented as mean ± standard deviation _(_$\bar{x}\pm \text{s}$). Categorical variables were analyzed using χ^2^ test or Fisher exact probability test, and continuous variables were analyzed using variance analysis of repeated measurement data. *P* < 0.05 was considered to be statistically significant.

## Result

As shown in Table 1, there were three men and seven women in group S, five men and five women in group M, four men and six women in group L. On admission of neuropathic pain, there were no significant differences in sex, age, BMI, and disease types among the three groups (Table 1). In addition, no significant difference was noted regarding the comorbidity, including coronary heart diseases, hypertension, and chronic gastritis.

**Table 1:** Comparison of the general clinical and demographic information among patients on admission.

	**Group S**	**Group M**	**Group L**	***P*-value**
Age(year)^a^	56.6 ± 16.7	58.7 ± 13.3	55.8 ± 14.9	0.39
Sex(M/F)	3/7	5/5	4/6	0.66
BMI (kg/m^2^)^a^	23.3 ± 4.7	24.9 ± 4.1	23.8 ± 5.2	0.54
Comorbidities				
coronary heart disease	1	0	1	0.43
hypertension	2	1	2	0.77
chronic non-atrophic gastritis	2	1	1	0.76
cervical spondylotic radiculopathy	2	1	2	0.77
lumbar disc herniation	1	3	2	0.52
herpes zoster or postherpetic neuralgia	7	6	6	0.86

a *Data are presented as means ± SD*.

As shown in Table 2, there was no significant difference in VAS among the three groups at T_0_. VAS was decreased continuously during the injection as well as after the injection. At the time D_2_, the VAS in group S was 5.25 ± 1.53 which was significantly higher than that in group M (3.89 ± 1.26, *P* = 0.033) and group L (3.79 ± 1.32, *P* = 0.028). At the time of W_4_, the VAS in group S (2.34 ± 0.89) was also higher than that in group M (1.58 ± 0.56, *P* = 0.043) and group L (1.43 ± 0.57, *P* = 0.036). No significant difference was noted at other time points.

**Table 2:** Comparison of VAS during and after the injection.

**Group**	**T_**0**_**	**D_**2**_**	**D_**4**_**	**D_**6**_**	**D_**8**_**	**D_**10**_**	**W_**1**_**	**W_**2**_**	**W_**3**_**	**W_**4**_**
S	8.23 ± 1.05	5.25 ± 1.53	4.41 ± 1.79	3.38 ± 1.76	2.88 ± 1.39	2.09 ± 1.03	1.23 ± 1.01	1.56 ± 1.39	1.88 ± 0.77	2.34 ± 0.89
M	8.17 ± 0.99	3.89 ± 1.26^*^	3.55 ± 1.58	3.00 ± 1.78	2.57 ± 1.59	1.89 ± 0.99	1.45 ± 0.88	1.44 ± 0.96	1.06 ± 0.89	1.58 ± 0.56^*^
L	8.09 ± 1.17	3.79 ± 1.32^*^	3.09 ± 2.01	2.77 ± 1.97	2.45 ± 1.33	1.57 ± 1.11	0.89 ± 0.93	1.37 ± 1.13	1.09 ± 0.86	1.43 ± 0.57^*^

*T_0_, time point before injection; D_2_, time point the 2nd day during the injection; W_1_, time points 7 days after injection; D, day; W, week; VAS, Visual Analog Score*.

*Data are presented as means ± SD*.

compared with Group S

* *P < 0.05*.

As shown in Table 3, there was no significant difference in FBG among the groups at T_0_. During the treatment, the FBG in group L was statistically higher than that in the groups S and M (*P* < 0.05), whereas there was no significant difference between group S and group M. At D_10_, FBG was higher than that at T_0_ in group L (*P* < 0.05). The level of 2hPG was higher during the treatment than that in the T_0_ (*P* < 0.05), whereas there was no significant difference after stopping the injection in the three groups.

**Table 3:** Comparison of FBG and 2hPG during and after the injections (mmol/L).

	**Group**	**T_**0**_**	**D_**2**_**	**D_**4**_**	**D_**6**_**	**D_**8**_**	**D_**10**_**	**W_**1**_**	**W_**2**_**	**W_**3**_**	**W_**4**_**
FBG	S	5.25 ± 0.67	4.98 ± 1.03^*^	5.11 ± 0.98^*^	4.79 ± 0.88^*^	5.38 ± 0.93^*^	5.66 ± 0.99^*^	5.27 ± 0.67	5.21 ± 0.59	5.48 ± 0.71	5.71 ± 0.66
	M	5.44 ± 0.69	4.99 ± 1.16^*^	5.53 ± 1.03^*^	4.96 ± 1.01^*^	5.26 ± 0.96^*^	5.48 ± 1.01^*^	5.37 ± 0.78	5.79 ± 0.66	5.18 ± 0.69	5.36 ± 0.72
	L	5.38 ± 0.71	6.56 ± 1.31	6.39 ± 1.35	6.09 ± 1.17	6.57 ± 1.03	6.88 ± 1.11 ^#^	5.72 ± 0.91	5.43 ± 0.73	5.69 ± 0.56	5.29 ± 0.88
2hPG	S	6.44 ± 2.03	7.11 ± 2.53 ^#^	7.23 ± 2.33 ^#^	7.84 ± 2.09 ^#^	7.22 ± 2.69 ^#^	7.01 ± 1.99 ^#^	6.24 ± 1.67	5.68 ± 2.09	6.09 ± 1.89	6.39 ± 2.16
	M	6.39 ± 2.19	7.25 ± 1.49 ^#^	7.36 ± 2.25 ^#^	7.69 ± 1.88 ^#^	7.58 ± 2.51 ^#^	7.63 ± 2.22 ^#^	6.38 ± 1.78	6.37 ± 2.26	6.28 ± 1.96	6.91 ± 2.28
	L	6.57 ± 2.31	7.68 ± 1.77 ^#^	7.85 ± 2.03 ^#^	7.85 ± 2.17 ^#^	7.99 ± 2.33 ^#^	8.56 ± 2.04 ^#^	6.71 ± 1.91	6.66 ± 2.57	6.19 ± 2.55	6.28 ± 2.88

*T_0_, time point before injection; D_2_, time point the 2nd day during the injection; W_1_, time points 7 days after injection; D, day; W, week; FBG, fasting blood glucose; 2hPG, 2 h postprandial blood glucose*.

*Data are preseted as means ± SD*.

compared with Group L

* P < 0.05; compared with T_0_

# *P < 0.05*.

During injection, the concentrations of cortisol and ACTH were significantly lower compared to T_0_, but they were gradually recovered to the normal concentration after stopping the injection (Table 4).

**Table 4:** Comparison of COR (nmol/L) and ACTH (pg/mL) during and after the injections.

	**Group**	**T_**0**_**	**D_**2**_**	**D_**4**_**	**D_**6**_**	**D_**8**_**	**D_**10**_**	**W_**1**_**	**W_**2**_**	**W_**3**_**	**W_**4**_**
COR	S	319.6 ± 75.2	44.1 ± 19.7^*^	39.5 ± 22.6^*^	30.2 ± 20.9^*^	31.7 ± 27.5^*^	28.8 ± 11.4^*^	296.3 ± 71.6	347.9 ± 92.0	329.9 ± 89.4	351.2 ± 96.3
	M	255.3 ± 89.9	38.3 ± 21.7^*^	30.3 ± 15.8^*^	29.1 ± 18.8^*^	33.3 ± 19.8^*^	27.9 ± 17.7^*^	319.2 ± 98.3	299.6 ± 77.6	373.6 ± 69.1	341.8 ± 84.4
	L	286.8 ± 79.4	35.2 ± 17.7^*^	28.1 ± 18.1^*^	27.6 ± 25.1^*^	29.4 ± 23.4^*^	27.8 ± 20.3^*^	277.1 ± 85.1	337.4 ± 79.3	360.5 ± 99.4	31.2 ± 93.2
ACTH	S	14.23 ± 5.23	6.78 ± 3.35^*^	5.48 ± 2.76^*^	5.22 ± 1.93^*^	5.28 ± 2.06^*^	5.10 ± 1.64^*^	8.72 ± 3.67	13.66 ± 3.52	13.42 ± 4.24	17.30 ± 3.56
	M	15.37 ± 6.96	6.23 ± 2.72^*^	5.21 ± 3.55^*^	5.76 ± 2.38^*^	5.32 ± 1.98^*^	5.06 ± 2.17^*^	10.67 ± 4.13	14.14 ± 3.75	14.19 ± 3.86	11.36 ± 4.70
	L	17.94 ± 7.91	5.85 ± 3.76	5.39 ± 2.24^*^	5.11 ± 2.51^*^	5.09 ± 1.70^*^	5.07 ± 2.09^*^	11.0 ± 4.86	16.23 ± 4.09	12.36 ± 4.11	13.28 ± 3.74

*T_0_, time point before injection; D_2_, time point the 2nd day during the injection; W_1_, time points 7 days after injection; D, day; W, week; COR, cortisol; ACTH, adrenocorticotropic hormone*.

*Data are presented as means ± SD*.

compared with T_0_

* *P < 0.05*.

As shown in Table 5, there was no significant difference in CHOL and TG among the three groups at each time point.

**Table 5:** Comparison of CHOL and TG during and after the injections (mmol/L).

	**Group**	**T_**0**_**	**D_**2**_**	**D_**4**_**	**D_**6**_**	**D_**8**_**	**D_**10**_**	**W_**1**_**	**W_**2**_**	**W_**3**_**	**W_**4**_**
CHOL	S	4.37 ± 1.11	5.01 ± 1.04	4.44 ± 0.96	4.69 ± 0.91	4.99 ± 0.86	4.96 ± 0.97	5.24 ± 0.76	5.21 ± 0.96	5.10 ± 0.88	4.39 ± 0.92
	M	4.88 ± 0.88	4.89 ± 1.12	4.36 ± 0.89	4.84 ± 1.30	5.19 ± 0.79	5.65 ± 1.08	4.38 ± 0.93	4.70 ± 0.70	4.28 ± 1.13	3.91 ± 0.84
	L	4.59 ± 0.99	5.08 ± 1.38	4.85 ± 0.75	5.26 ± 0.55	4.78 ± 1.12	4.56 ± 1.04	4.71 ± 0.85	4.66 ± 0.65	4.09 ± 1.00	4.28 ± 1.05
TG	S	1.01 ± 0.29	0.93 ± 0.38	0.87 ± 0.33	1.23 ± 0.50	1.24 ± 0.22	1.39 ± 0.39	1.24 ± 0.25	1.32 ± 0.19	1.31 ± 0.36	1.33 ± 0.40
	M	1.04 ± 0.35	1.08 ± 0.38	0.96 ± 0.39	0.95 ± 0.37	1.23 ± 0.34	1.06 ± 0.41	1.38 ± 0.28	1.45 ± 0.22	1.28 ± 0.34	1.28 ± 0.22
	L	0.99 ± 0.30	1.21 ± 0.44	0.98 ± 0.41	0.89 ± 0.30	1.11 ± 0.31	1.35 ± 0.30	1.03 ± 0.33	0.98 ± 0.32	1.32 ± 0.42	1.27 ± 0.34

*T_0_, time point before injection; D_2_, time point the 2nd day during the injection; W_1_, time points 7 days after injection; D, day; W, week; CHOL, total cholesterol; TG, triglyceride*.

*Data are presented as means ± SD*.

*There was no significant difference in CHOL and TG among the three groups at each time point*.

### Adverse Reactions

None of the patients developed nausea, vomiting, urinary retention, limb weakness, catheter detachment, catheter fracture, and catheter-related infections during and after the injection. Two patients from groups S and M had mild pruritus with self-relieving. One patient from group L had insomnia, and the symptom disappeared after the oral administration of diazepam.

## Discussion

In this study, we found that continuous epidural injection can effectively alleviate pain among patients with cervical spondylotic radiculopathy, lumbar disc herniationor herpes zoster and postherpetic neuralgia (except cranial nerve). The concentrations of cortisol and ACTH were recovered to the normal level after stopping the injection. The treatment did not affect CHOL and TG. No serious adverse reactions occurred during the study.

ESI was first recorded by Lievre and his colleagues in 1953. However, rare but serious adverse reactions have been reported (13), such as chemical meningitis and spinal cord embolism. Studies have shown that these adverse reactions are related to the type and particle size of glucocorticoids (14, 15). Most of these adverse reactions occur in the use of drugs composed of large particles such as triamcinolone acetonide and methylprednisolone acetate. Studies have shown that the molecular size of soluble dexamethasone is about 1/10 of the volume of red blood cells (16). Therefore, dexamethasone has become a safe drug for ESI. The advantage of ESI is that a lower dose of corticosteroids be injected into the epidural space closest to the affected nerve roots, which can rapidly eliminate inflammation and edema caused by intervertebral disc degeneration or viral damage, and improve neurological ischemia, avoid central sensitization (17).

Our data showed that the analgesic effect in group S was slightly poorer at the initial stage of treatment compared to other groups, but the analgesic effect was largely similar at other time points, suggesting that continuous epidural injection of dexamethasone with a dose of 50 μg/mL might reach a satisfactory analgesic effect.

In addition, our data showed that fasting blood glucose in group L was statistically higher than that in group M and group S, but they gradually recovered after stopping the injection. 2hPG was higher during the injection probably due to glucocorticoids promoting gluconeogenesis in the liver and muscle and inhibiting the uptake and utilization of glucose by peripheral tissues. Xu and his colleagues (18) found that a single injection of 10 mg dexamethasone would increase the FBG temporarily. Mohamed Younes et al. (6) have studied 18 patients withthree injections of 1.5 mL cortivazol in epidural space, and showed no significant increase in fasting blood glucose, but significantly elevated blood glucose,. These results suggested that it was safe to inject an appropriate dose of glucocorticosteroid into the epidural space, although it can affect blood glucose temporarily. It should be cautious for patients with diabetes to receive the continuous epidural injection and frequent monitor of the blood glucose is highly recommended.

Long-term systemic application of glucocorticoids can cause hyperlipidemia, but little is known about the effect of ESI on blood lipids. Only one study showed that serum triglycerides and cholesterol increased significantly after 1 week of treatment of high-dose prednisone (19). However, our data suggested that CHOL and TG were still kept at the normal levels during and after the treatment, even for patients who received a continuous epidural injection of 100 μg/mL dexamethasone.

Cortisol is a steroid hormone that is released by cells of the adrenal cortex (20). Its secretion is highest in the morning and the lowest at midnight (12). In our study, we measured the concentration of cortisol and ACTH at 8:00 am, which could better reflect the HPAA status. Maillefert et al. (21) showed a HPAA suppression <3 weeks after a single epidural injection of 15 mg dexamethasone. Abdul et al. (22) have indicated that HPAA function was suppressed after the ESI of 80 mg methylprednisolone acetate for 14 days, and returned to the normal range 4 weeks later. Choon et al. (23) studied the salivary cortisol concentrations after a single ESI of 40 mg triamcinolone, which showed that HPAA suppression was observed in all patients after ESI. George Habib et al. (8) showed that both serum cortisol and ACTH were significantly lower compared with baseline levels in all patients, but this suppression was transient.

It should be noted that this study has several limitations when we interpret the findings. First, the participants in this study were relatively small, which calls for further studies to confirm our research findings. Second, we didn't study the effect on blood pressure and cardiac diseases after the treatment which should be addressed in further studies. The advantages of this study include its prospective study design, a fine methodology, and relatively appropriate endpoints.

In conclusion, our data suggested that continuous epidural injection of 50 μg/mL dexamethasone can achieve a satisfactory analgesic effect and less adverse effect on blood glucose, plasma cortisol and ACTH.

## Data Availability Statement

The raw data supporting the conclusions of this article will be made available by the authors, without undue reservation.

## Ethics Statement

The studies involving human participants were reviewed and approved by The Second Hospital of Hebei Medical University. The patients/participants provided their written informed consent to participate in this study.

## Author Contributions

All authors contributed to the article and approved the submitted version.

## Conflict of Interest

The authors declare that the research was conducted in the absence of any commercial or financial relationships that could be construed as a potential conflict of interest.
